# Genomic complexity in pediatric synovial sarcomas (Synobio study): the European pediatric soft tissue sarcoma group (EpSSG) experience

**DOI:** 10.1002/cam4.1415

**Published:** 2018-03-13

**Authors:** Daniel Orbach, Véronique Mosseri, Daniel Pissaloux, Gaelle Pierron, Bernadette Brennan, Andrea Ferrari, Frederic Chibon, Gianni Bisogno, Gian Luca De Salvo, Camille Chakiba, Nadège Corradini, Véronique Minard‐Colin, Anna Kelsey, Dominique Ranchère‐Vince

**Affiliations:** ^1^ SIREDO oncology center (Care, Innovation and Research for Children, Adolescents and Young Adults with cancer) Institut Curie PSL university Paris France; ^2^ Department of Biostatistics Institut Curie Paris France; ^3^ Biopathology Department Institut d'Hematologie et d'Oncologie Pediatrique Centre Léon Bérard Lyon France; ^4^ Somatic Genetic Unit Institut Curie Paris France; ^5^ Department of Paediatric Oncology Royal Manchester Children's Hospital Manchester UK; ^6^ Pediatric Oncology Unit Fondazione IRCCS Istituto Nazionale Tumori Milano Italy; ^7^ Département de Biopathologie Institut Bergonié Bordeaux Cedex France; ^8^ Pediatric Hematology and Oncology Division Padova University Padova Italy; ^9^ Clinical Trials and Biostatistics Unit IRCCS IstitutoOncologico Veneto Padova Italy; ^10^ Institut d'hématologie et d'Oncologie Pédiatrique Centre Léon Bérard Lyon France; ^11^ Department of Paediatric and Adolescent Oncology Gustave‐Roussy Villejuif France; ^12^ Department of Diagnostic Paediatric Histopathology Royal Manchester Children's Hospital Manchester UK

**Keywords:** Adolescent, comparative genomic hybridization, EpSSG, genomic index, synovial sarcoma

## Abstract

A genomic index (GI) tool using array comparative genomic hybridization (aCGH) on tumor cells has emerged as independent prognostic factor associated with the risk of metastatic relapse in synovial sarcoma (SS). The aim was to assess GI in pediatric patients with SS, to determine its value as a prognostic factor. All pediatric/adolescent/young adults’ (<25 years) with localized SS prospectively included in the European EpSSG‐NRSTS05 protocol with a contributive aCGH were selected. Definition of GI was *A* ^2^/*C*, where *A* is the total number of alterations (segmental gains and losses) and C is the number of involved chromosomes on aCGH results. GI_1_ group corresponds to cases with no copy number alterations (flat profile, GI = 0) and GI_2_ group cases with at least one or more copy number alterations (rearranged profile; GI ≥ 1). Samples were available from 61 patients. The median age of the cohort was 13 years (range: 4–24). Overall, 55.7% were GI_1_ group, and 44.3% GI_2_. After a median follow‐up of 62 months (range: 0.1–112), 10 tumor events occurred and five patients died. Respectively, for GI_1_ versus GI_2_ groups, five‐year event‐free survival (EFS) was 93.8 ± 4.2% versus 64.9 ± 10.1% (*P* < 0.006) and five‐year Metastatic‐Free Survival (MFS) 93.8 ± 4.2% versus 72.9 ± 9.5% (*P* < 0.04). In multivariate analysis, GI status as adjusted for IRS group, patient age, site, and tumor size remain independent prognostic for EFS with a relative risk (RR) of 6.4 [1.3–31.9] (*P* < 0.01) and RR for MFS is 4.8 [0.9–25.7] (*P* < 0.05). Genomic complexity evaluated through GI may explain the metastatic behavior of pediatric SS.

## Introduction

Synovial sarcoma (SS) is a malignant mesenchymal tumor that occurs in both pediatric and adult age. It accounts for 8–10% of all soft tissue sarcomas (STS) in children. The overall median age at diagnosis is 32 years, with 30% of SS occurring before 20 years of age (most of them in adolescence; median age 13.7 year) 1, 2. The prognosis depends mainly on the feasibility of surgical resection and the tumor's size and site, and the presence of metastases, but the optimal treatment remains to be fully ascertained 3, 4, 5. Recent improvements in knowledge of the biology of SS may enable new biological markers to be identified and applied to patient selection, thereby improving prognostic accuracy and the efficacy of therapies. A 67‐gene signature related to chromosome integrity and genome complexity named CINSARC (complexity index in sarcoma), or a genomic index (GI) analyzed using comparative genomic hybridization on tumor cells, have recently been developed and shown a high prognostic value in STS 6, 7, 8, 9. CINSARC and GI have also emerged as independent prognostic factors associated with the risk of metastases developing in adult and pediatric SS 10. Differences in genome complexity have been observed between adult and in some pediatric cases. In this series, among the 21 pediatric patients analyzed, the two cases of SS that metastasized were associated with a high GI 10, 11. When the feasibility of adapting the indication for chemotherapy to the tumor's molecular profile was further explored in another cohort of patients, the study confirmed that complex somatic molecular abnormalities were associated with outcome, but found no correlation with response to neoadjuvant chemotherapy 12. In other words, GI does not seem to be associated with the tumor's chemosensitivity, but it may reveal intrinsic biological characteristics that could be used to stratify patients for their risk of future metastases, and this could point to the need for more intensive therapy in some cases. The NRSTS 2005 protocol (Non Rhabdomyosarcoma STS Study) included a prospective nonrandomized trial on SS for the purpose of assessing the role of full‐dose ifosfamide‐doxorubicin chemotherapy in improving the response rates of patients with unresectable disease and examining the impact of omitting adjuvant chemotherapy in low‐risk cases. This EpSSG trial (European pediatric STS Group), involving 138 patients <21 years old with SS, includes 15 different countries, with 131 centers in all 2, 13. This report showed satisfactory overall results (with 5‐year EFS and OS rates of 80.7 and 90.7%, respectively) encouraging us to determine who needs chemotherapy which might be better predicted by recent findings on somatic genomic abnormalities.

The main aim of this project was therefore to analyze the aCGH value (array Comparative Genomic Hybridization) of the tumors from already published patients in order to determine, in a larger set of pediatric and adolescent patients, if GI could be used as a prognostic factor and help to better stratify patients risk for the future protocol. Additional goal is to determine whether genomic instability may define a genotype‐phenotype correlation in SS.

## Material and Methods

### Inclusion criteria

All pediatric and adolescent patients (<25 year) with initially localized SS, prospectively registered in the EpSSG NRSTS 05 protocol in agreement of families and patients already obtained at baseline by signature during the initial protocol inclusion were selected. Tumors sample did have a centralized pathology review and enough tumor material to have an aCGH retrospectively analyzable 14. This study included patients from 2005 to 2012 and was conducted according to the agreements of the Declaration of Helsinki and Good Clinical Practice and the European Union Directive 2001 statement regarding/20/EC for noncommercial clinical trials (European Union Drug Regulating Authorities Clinical Trials No. 2005‐001139‐31) 2.

In the risk‐adapted EpSSG NRSTS‐2005 trial for synovial sarcoma, low‐risk patients (complete resection R0/IRS‐I, with tumor <5 cm; limbs primary) were treated with surgery alone (no adjuvant therapy) 13; intermediate‐risk patients (complete resection with tumor >5 cm, or microscopic resection R1/IRS‐II; limbs) had three to six courses of adjuvant ifosfamide‐doxorubicin based chemotherapy ± radiotherapy; high‐risk patients (incomplete macroscopic resection or biopsy R2/IRS‐III; or axial primary) had six courses of chemotherapy, delayed surgery (when feasible), and radiotherapy (local treatment had to be planned after three cycles of neoadjuvant chemotherapy). The main chemotherapy regimen was ifosfamide 3 g/m²/day, for 3 days + doxorubicin 37.5 mg/m²/day, for 2 days. Two cycles of ifosfamide 3 g/m²/day for 2 days concomitantly to radiotherapy was added in high‐risk group. Radiotherapy doses in IRS‐III tumors are 59.4 Grays (Gy) without the option of secondary resection; 50.4 Gy as preoperative radiotherapy; 50.4, 54.0, and 59.4 Gy as postoperative radiotherapy, in the case of R0, R1, and R2 resections, respectively. Clinical staging was defined according to the tumor node metastases system: T1 or T2 according to the invasion of contiguous organs; N0/N1, according to the presence of lymph node.

### Experimental procedures

**Figure 1:**
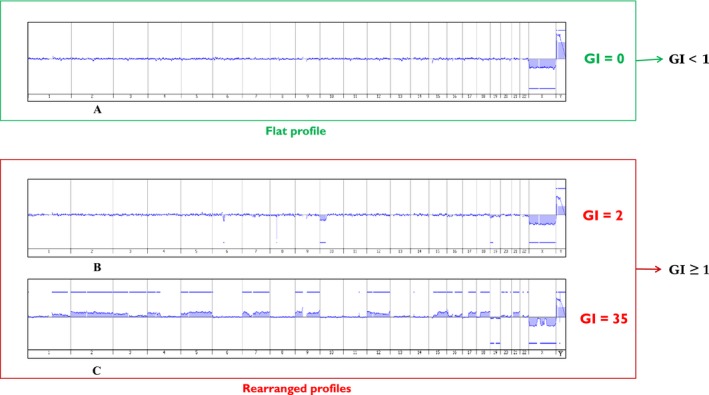
Examples of different synovial sarcoma aCGH profiles: without any alteration (A), slightly rearranged (B), and highly rearranged (C).

Genomic DNA was extracted from paraffin‐embedded tumors according to Agilent protocol for DNA isolation on formalin‐fixed, paraffin‐embedded (FFPE) tissues (Agilent Technologies). DNA was then treated using a DNase and hybridized to 4x180K whole‐genome Agilent arrays (G4449A) as previously described 10, 12. Microarray slides were scanned using an Agilent DNA microarray scanner; raw images were treated by Feature Extraction V11.5.1.1 and then analyzed by Agilent Genomic Workbench V7.0.4.0 (Agilent). The ADM‐2 algorithm was used to identify DNA copy‐number anomalies at the probe level. A copy‐number gain was defined as a log2 ratio >0.25 and a copy‐number loss as a log2 ratio ≤0.25. The GI is calculated and applied for each profile: GI = *A* ^2^/*C*, where *A* is the total number of alterations (segmental gains and losses) and *C* is the number of involved chromosomes 12. Profiles are sorted into two different groups: cases where no alterations were present (GI_1_ group with flat aCGH profile; GI = 0) corresponded to the low GI group, cases presenting many alterations (GI_2_ group with rearranged aCGH profile; GI ≥ 1) formed the high GI group (Fig. 1). The percent of tumor cells in each sample were analyzed and should be at least 50%.

### Statistical methods and data collection

Data were analyzed considering information within the Remote Data Entry system at March 2017. Outcome was defined as overall survival (OS), event (EFS) and metastatic‐free survival (MFS). The definition of OS was measured from the date of diagnosis to death from any cause. Events were defined for EFS as progression during chemotherapy, relapse after complete remission (CR), or death from any cause. MFS was calculated by the Kaplan–Meier method from the date of initial diagnosis to the date of first metastasis, last follow‐up, or death for all patients without diagnosis of metastasis. Survival curves were calculated by the Kaplan–Meier method. The five‐year rates were expressed together with their standard error. For univariate analysis, the statistical significance of each variable was first tested by the log‐rank test. Multivariate analysis was then performed with the Cox proportional hazards models for MFS and EFS: The aim was to assess the GI's prognostic value adjusted for tumor size, tumor site, and IRS group, already known as risk factors. In addition, patient age was analyzed. The group risk was not introduced in the model studying MFS, as there was a problem of statistical convergence, no metastasis occurring in the low or intermediate group. For homogeneity, it was not introduced either in the model studying EFS. No backward or stepwise procedure was performed. The multivariate analysis model's *P* values were determined using the likelihood ratio test.

## Results

**Figure 2:**
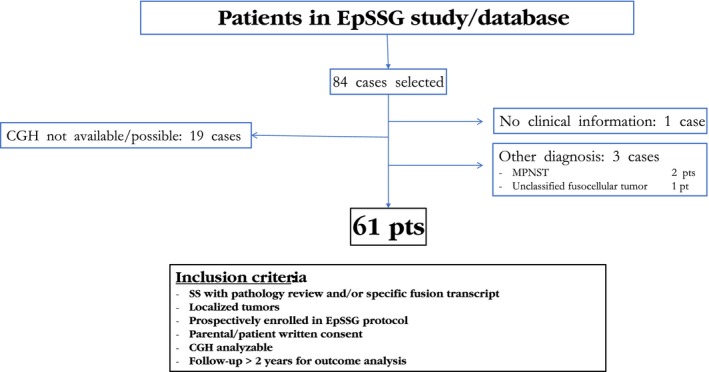
Flowchart of the study. SS, synovial sarcoma; pt: patient; NOS, nonother specification; MPNST, malignant peripheral nerve sheath tumor.

Among a total of 84 patients, of 213 patients registered in the protocol in the same period, with tumor sample available, 61 cases reach inclusion criteria for this study. Patients were treated in five European countries (France, UK, Italy, Spain, and Norway). Population selected and tumor characteristics are indicated in Table 1. Among them, 95% harbored one of the characteristics transcripts and 95% had a pathology review. Overall, 23 cases were not included, due to nonconfirmed diagnosis (three cases), the absence of clinical data (1 case) or aCGH not possible (not enough tumor material 10 cases, the absence of available tumor sample eight cases, and technical problem one case) (Fig. 2).

**Table 1:** Patients and tumor characteristics

Initial characteristics	Numbers of patients 61 cases	%
Median age (ranges)	13 years (4–24)	
Male	38	
Female	23	
Primary		
Limbs	42	69
Trunk	12	20
Head and neck	7	11
Tumor size (>5 cm)	28	46
TNM		
T1	51	83
T2	9	15
Unknown	1	2
IRS groups		
IRS‐I	22	36
IRS‐II	13	21
IRS‐III	26	43
Risk group		
Low risk	13	21
Intermediate risk	10	17
High risk	38	62
Histology subtypes		
Monophasic	46	76
Biphasic	13	21
Unknown	2	3
FNCLCC grading		
Grade 2	41	67
Grade 3	11	18
Unknown	9	15
Transcript		
SSX1	30	49
SSX2	9	15
One of both	19	32
Negative	1	1
Not performed	2	3
Genomic index		
Low	34	56
High	27	44

FNCLCC, Federation Nationale des Centres de LutteContre le Cancer;IRS I, complete resection; IRS I, microscopic residue; IRS III, macroscopic residue; R0, complete delayed surgery; R1, microscopic incomplete delayed surgery; R2, macroscopic incomplete delayed surgery. T1, tumor localized in the organ or origin; T2, tumor extend beyond organ or tissue of origin.

**Figure 3:**
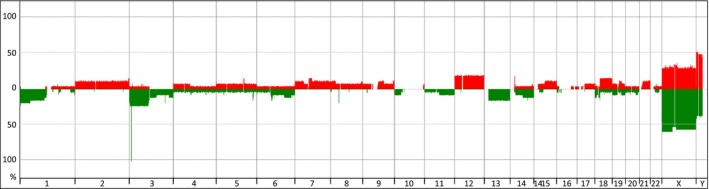
Penetrance plot of GI_2_ synovial sarcomas analyzed with aCGH (27 cases).

These tumors occur most commonly in adolescents (median age at diagnosis: 13 years) and limbs primary (69%). Overall, 62% of cases were classified as high risk, and 43% of IRS‐III tumors. Monophasic pathology was the most frequent histological subtype (76%) with a majority of FNCLCC (*Fédération Nationale des Centres de Lutte contre le Cancer* grading system) grade 2 tumors (67%). Patients with tumor harboring a high GI represented 44% of the population (Table 1). Median tumor cells in samples with flat profiles were 70% (range, 50–70%). Penetrance plot for tumors with high GI showed some losses of 1p, 3p and chromosome 13 associated to gain of chromosome 12 and 18q (Fig. 3). Comparison between the two populations with high and low GI found no difference according to patients and initial tumor characteristics, even if tumors with high GI have a trend to be more extensive (T2), and in higher risk groups (Table 2). High GI was present in 19/41 (46%) FNCLCC grade II, 6/11 (54%) grade III and 2/9 (22%) unknown grade tumors. Overall, 13 patients had surgery only (low risk), 10 received adjuvant therapy after surgery (intermediate risk), and 38 had a perioperative chemotherapy associated to local therapy (high‐risk group) according to protocol.

**Table 2:** Patients and tumors characteristics according to the genomic index

	Low GI 34 pts	High GI 27 pts	*P* value
Age (median)	12 years	13 years	0.49
Male/female	22/12	16/11	0.66
Primary			
Limbs	76.5%	59.3%	0.15
Trunk + Head Neck	23.5%	40.7%	
Tumor size			
≤5 cm	58.8%	48.2%	0.41
>5 cm	41.2%	51.8%	
TNM			
T1	93.9%	74.1%	0.06
T2	6.1%	25.9%	
IRS groups			
IRS‐I	38.2%	33.3%	0.36
IRS‐II	26.5%	14.8%	
IRS‐III	35.3%	51.9%	
Risk group			
Low risk	29.4%	11.1%	0.09
Intermediate risk	20.6%	11.1%	
High risk	50.0%	77.8%	
Histology subtypes			
Monophasic	75.7%	80.8%	0.65
Biphasic	24.3%	19.2%	
FNCLCC grading			
Grade 2	81.5%	76.0%	0.63
Grade 3	18.5%	24.0%	
Five‐year event rate	6.2% [0–14.4]	35.1% [15.4–54.9]	<0.006
Five‐year metastatic rate	27.1% [8.6–45.7]	6.2% [0–14.4]	<0.04

**Figure 4:**
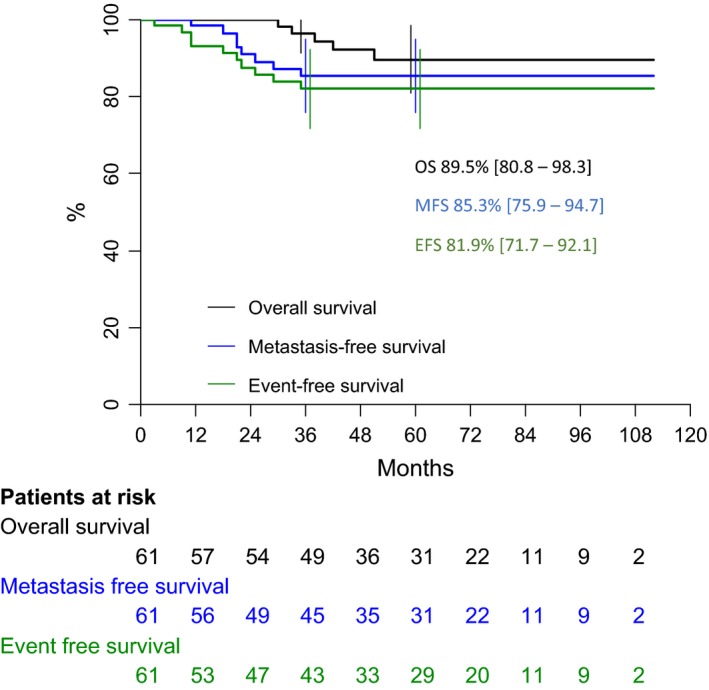
Overall outcome of all patients with synovial sarcoma. OS, overall survivals; EFS, event‐free survival; MFS, metastatic‐free survival.

**Figure 5:**
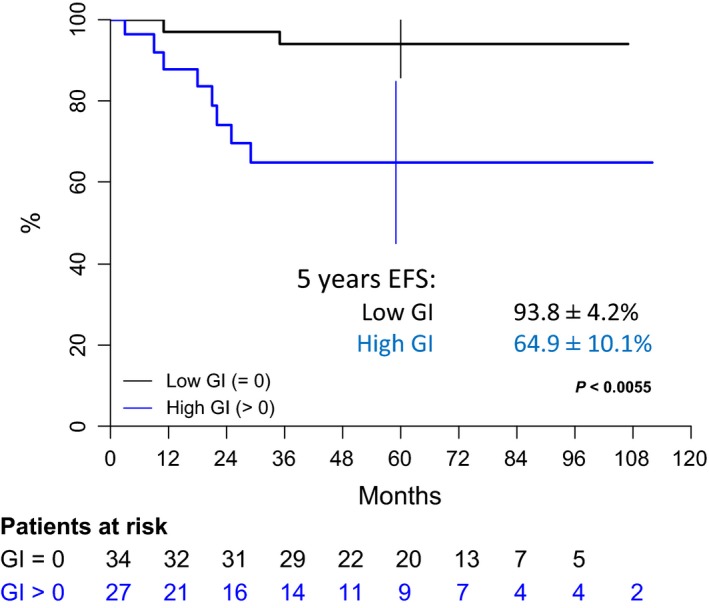
Event‐free survival of patients with localized synovial sarcoma according to the genomic index value. EFS, event‐free survival; GI, Genomic index.

**Figure 6:**
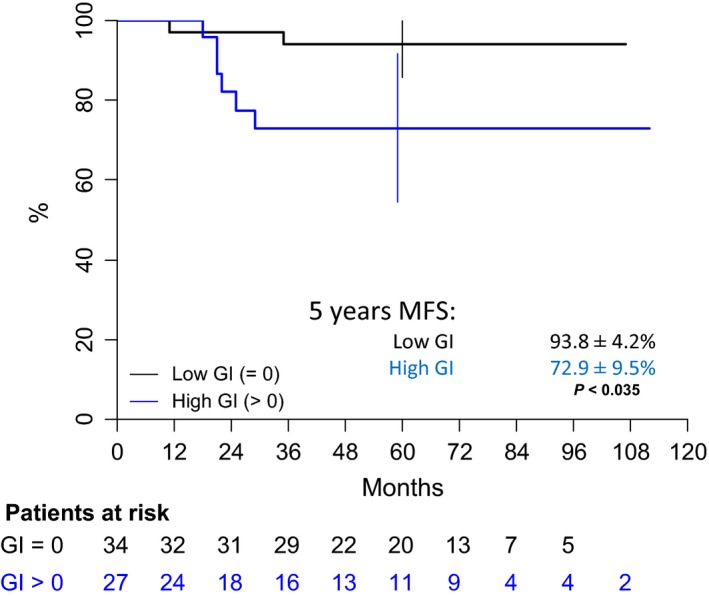
Metastatic‐free survival of patients with localized synovial sarcoma according to the genomic index value. MFS, metastatic‐free survival; GI, Genomic index.

After a median follow‐up of 62 months (range 1–112), 10 tumor events occurred, 3–35 months after diagnosis: local progressive disease (one case), local relapse (one case), combined (local + metastatic) relapse (four cases), and isolated metastatic relapse (four cases). At the end of the follow‐up, the eight distant metastatic relapses led to five deaths despite salvage therapy, 30–51 months after diagnosis. Five‐year OS, MFS, and EFS of the overall population are therefore, respectively, 89.5% [80.8–98.3], 85.3% [75.9–94.7], and 81.9% [71.7–92.1] (Fig. 4). Patients with low GI tumors have a favorable outcome in comparison with patients with high GI tumors and present less overall events and less metastatic tumor events with a five‐year EFS 93.8 ± 4.2% versus 64.9 ± 10.1% (*P* < 0.006; Fig. 5, Table 2) and a MFS of 93.8 ± 4.2% versus 72.9 ± 9.5% (*P* < 0.04; Fig. 6). Univariate analysis shows that both group risk and GI have an impact on MFS and EFS (Table 3), whereas in multivariate analysis, GI status as adjusted for IRS group, site, patient age, and tumor size remains independent prognostic for EFS, with a relative risk (RR) of 6.4[1.3–31.9] (*P* < 0.01), and for MFS (RR 4.8 [0.9–25.7]; *P* < 0.05) very close to the univariate estimations. OS was 96.9 ± 3.1% versus 78.8 ± 9.5%, respectively, for patients with low GI and high GI (*P* = 0.06).

**Table 3:** Univariate and multivariate analysis with relative risk of GI status adjusted for site, size, and IRS‐group for patients with synovial sarcoma

Univariate analysis				
Risk factors	5‐year MFS	*P* value	5‐year EFS	*P* value
Age at diagnosis				
≤10 years	94.1 ± 5.7%	0.21	88.5 ± 7.6%	0.38
≥11 years	81.2 ± 6.4%		78.8 ± 6.6%	
Group risk				
LR + IR	100%	0.01	95.5 ± 4.4%	<0.04
HR	75.3 ± 7.6%		72.7 ± 7.8%	
IRS group				
IRS I	95.5 ± 4.6%	0.26	90.5 ± 6.4%	0.39
IRS II	80.8 ± 12.2%		82.5 ± 11.3%	
IRS III	78.4 ± 8.6%		74.3 ± 9.1%	
Tumor size				
≤5 cm	92.9 ± 4.9%	0.09	89.8 ± 5.8%	0.11
>5 cm	77.3 ± 8.2%		73.9 ± 8.5%	
Tumor site				
Limbs	89.8 ± 4.8%	0.13	84.8 ± 5.7%	0.38
Axial/Head and Neck	73.7 ± 11.3%		74.3 ± 11.1%	
Genomic index				
Low	93.8 ± 4.2%	<0.04	93.8 ± 4.2%	<0.006
High	72.9 ± 9.5%		64.9 ± 10.1%	

Multivariate analysis				
	MFS Relative risk CI (0.95)	*P* value	EFS Relative risk CI (0.95)	*P* value
GI status				
=0	1.00	<0.04	1.00	<0.009
>0	5.3 [0.95–29.8]		6.7 [1.3–33.9]	
Site				
Axial/Head and Neck	1.0		1.0	
Limbs	0.3 [0.1–1.5]		0.6 [0.2–2.6]	
Tumor size				
<5 cm	1.0		1.0	
≥5 cm	1.3 [0.2–9.2]		1.5 [0.3–7.1]	
IRS group				
I	1.0		1.0	
II	3 [0.2–42.6]		2.0 [0.2–16.6]	
III	2.3 [0.2–25.9]		1.6 [0.3–9.7]	
Age at diagnosis				
≤10 years	1.0		1.0	
≥11 years	3.7 [0.3–42.2]		2 [0.3–12.4]	

OS, overall survivals; EFS, event‐free survival; MFS, metastatic‐free survival; HR, high risk; IR, intermediate risk; LR, low‐risk group; IRS‐I/R0, complete resection; IRS‐II/R1, microscopic residue; IRS‐III/R2, macroscopic residue or biopsy; CI, confidence interval.

## Discussion

This study in a large set of pediatric and adolescent patients with localized synovial sarcoma shows for the first time that despite the small number of tumor events after a multidisciplinary therapeutic strategy, tumor biology strongly influences outcome. General characteristics of the population of this study confirm that localized SS occurs mainly during adolescence in limbs and has an overall favorable outcome in young patients. As already described, remaining unfavorable features for patients included in the EpSSG are “high‐risk group” with initial unresectable tumor or an axial primary 2. As in other STS occurring during adult age, the standard treatment for localized SS remains surgery. There is still nevertheless no general agreement on the exact role of other combined treatments (radiotherapy and chemotherapy) specially in young patients 11. Different strategies have been adopted in the past for pediatric and adult groups, but the situation has now changed to some degree, and clinical approaches have tended to converge toward a shared strategy. The role of chemotherapy, however, in the setting of patients with localized disease is still to be determined 15. Recently, data have confirmed that in some favorable situations (complete resection of small localized tumors), no adjuvant therapy could be advocated in pediatric SS 13. In the aim to develop a risk‐adapted strategy, high GI could be added to the list of pejorative known risk factors in synovial sarcomas such axial primary site 3, 4, large tumors (>5–7 cm) 1, 3, 4, 16 with high FNCLCC histologic grade 16, 17, advanced stage at diagnosis 3, 17, 18, 19, 20, or type of SYT‐SSX fusion genes 21. This shift of an overall strategy in children and adolescent, in which all soft tissue sarcomas require systematic chemotherapy in better selected patients, could therefore be supported by biologic tools, such as the GI analysis. Several groups have performed genomic and gene expression profiling of soft tissue sarcomas and have identified diagnostic and prognostic signatures that characterize specific sarcoma subgroups. The aim of these studies was to try to identify molecular somatic markers that can better predict progression‐free survival. Different predictive biological molecular signatures have already been shown as effective in desmoid‐fibromatosis like various soft tissue sarcomas such as gastrointestinal stromal tumors (GIST), synovial sarcomas, uterine smooth muscle tumors with uncertain malignant potential (STUMP), and even other nonsarcoma tumors (breast carcinomas and lymphomas) 6, 7, 8, 9, 22, 23, 24, 25. These studies aimed to try to help clinicians to define patients with a high risk of tumor recurrence or a metastatic event that needs intensification of therapy and on the other hand reduced overall therapy in the cases with low‐risk characteristics. Chibon et al. 26 identified, validated, and confirmed recently a 67‐gene expression signature (CINSARC) that strongly correlates to metastasis free survival (MFS). The majority of the 67 CINSARC genes encode major regulators of cell cycle, immune check points, and chromosomal integrity. This gene expression signature assigns patient outcome better than the histological FNCLCC grading system and many other biological signatures 26.

The possible reasons why the genomic index reflects a more metastatic tumor are not known, but do not seem to be strictly related to the tumor chemosensitivity 10, 11, 12. The hypothesis is that, in some tumors, the more rearranged a genome is, the higher the probability to obtain a gene expression profile permitting cells to disseminate and develop distant metastases. Our study statistically failed to demonstrate that the biological characteristics may explain the published unfavorable clinical characteristics, such as axial primary, or a large tumor even if some trends exist (Table 2) 3, 4. Furthermore, the value of the aCGH profile complexity is currently recognized in some other pediatric tumors and used to stratify therapy, that is in infant and childhood neuroblastoma 27. Despite the overall good prognosis in pediatric SS, our study helps to select a subpopulation of patients who have a risk of metastatic event after diagnosis of 27.1% [8.6–45.7], despite conventional treatment, and will therefore require additional medical therapy in the future protocols with new drugs or maintenance therapy. In addition, prospective studies are warranted to propose medical therapy reduction for patients with low biological risk features.

Previous studies have shown that high GI is more frequent in the adult population with SS that has also poorer risk factors and a worse outcome 10, 12, 19, 28. In Lagarde et al. 10 experience, 64% of adults with synovial sarcomas have a rearranged profile and 76% of them (28/37 cases) developed a metastatic event. In comparison, they found that this unfavorable aCGH profile was present in only 19% of the pediatric patients (<18 year; 4/36 cases), in which 2/4 cases had metastases. In chakiba et al. 12 experience, rearranged profile was present in 56% of the 25 pediatric patients (<18 year; 14/25 cases). Our larger study showed that high GI was present in young patients with SS in 44% of the cases (<25 year; 27/61 cases). Notably, population selection was not strictly identical between these analyses as our study only selected pediatric patients with localized tumor at diagnosis, whereas Lagarde et al. and Chakiba et al. included patients with all stages of SS at diagnosis. Despite its unique and same translocation in SS, the clinical presentation and behavior of synovial sarcoma seem diverse across the ages. Treatment modalities seem to be different between these populations with more medical therapy delivered in children, but the impact of these different strategies remains to be determined 5, 10. Although the GI was not predictive of chemotherapy efficacy, Chakiba et al. 12 highlighted some genomic alterations that were significantly associated with overall response to chemotherapy, that is, gains in chromosomes 2 and 12 and losses in chromosomes 3 and 6 which are overrepresented in the group of patients with good and intermediate overall response.

Despite a large number of registered cases, suitable tumor samples could only be analyzed in a proportion of patients included in the European EpSSG protocol (61/138 cases) due to multiple reasons (small initial biopsies; noninformative aCGH; organizational). Further limitation of our study, is that, even if aCGH is a widely used biological tool, especially for the risk stratification analysis in pediatric neuroblastoma, harmonization of the GI technic is necessary to correctly analyze results. As commercial aCGH platforms can vary in resolution, design, and evaluation of both CNV (Copy Number Variation) and LOH (Loss Of Heterozygoty), detection of small chromosomic aberrations could modify the GI results if they are all considered. Finally, we should take into consideration that although the results of this series are in concordance with those already published by Lagarde, Chakiba et al.*,* our patients only had localized SS at diagnosis when their series included patients with both localized and metastatic tumors.

## Conclusion

Genomic complexity was significantly associated with the risk of metastasis and hence outcome in pediatric SS. Even, if the final OS showed not statistical difference due to the relative small number of patients in this series, this biological factor appears as the strongest prognostic factor in multivariate analysis. Given that the initial genetic driver event (the *t*(X;18) translocation) is usually present, this is likely to mean that an independent, still unknown mechanism leads to chromosome instability. Therefore, this study confirms that biology could help to better stratify patients with SS for future international European protocols using a relatively easy biological test at diagnosis. The GI score might be improved in the future, as it currently takes into account copy number alterations, which are only one aspect of the overall genome complexity. Here we have described two types of profiles, a rearranged one with high GI and a simple one with flat profiles. In this latter category, we might consider that other mechanisms are involved that explain the oncologic process. The evaluation of point mutations across the genome (mutation load) or other epigenetic makers could help to refine a better signature, with a better prognosis value.

## Conflict of Interest

All authors disclose any actual or potential conflict of interest including any financial, personal, or other relationships with other people or organizations within that could inappropriately influence (bias) their work.

## References

[1] Sultan, I. , C. Rodriguez‐Galindo , R. Saab , S. Yasir , M. Casanova , and A. Ferrari . 2009 Comparing children and adults with synovial sarcoma in the Surveillance, Epidemiology, and End Results program, 1983 to 2005: an analysis of 1268 patients. Cancer 115:3537–3547. 19514087 10.1002/cncr.24424

[2] Ferrari, A. , G. L. De Salvo , B. Brennan , M. M. van Noesel , A. De Paoli , M. Casanova , et al. 2014 Synovial sarcoma in children and adolescents: the European Pediatric Soft Tissue Sarcoma Study Group prospective trial (EpSSG NRSTS 2005). Ann. Oncol. 26:567–572. 25488687 10.1093/annonc/mdu562

[3] Orbach, D. , H. Mc Dowell , A. Rey , N. Bouvet , A. Kelsey , and M. C. Stevens . 2011 Sparing strategy does not compromise prognosis in pediatric localized synovial sarcoma: experience of the International Society of Pediatric Oncology, Malignant Mesenchymal Tumors (SIOP‐MMT) Working Group. Pediatr. Blood Cancer 57:1130–1136. 21495161 10.1002/pbc.23138

[4] Ferrari, A. , G. Bisogno , R. Alaggio , G. Cecchetto , P. Collini , A. Rosolen , et al. 2008 Synovial sarcoma of children and adolescents: the prognostic role of axial sites. Eur. J. Cancer 44:1202–1209. 18440800 10.1016/j.ejca.2008.03.016

[5] Ferrari, A. , A. Gronchi , M. Casanova , C. Meazza , L. Gandola , P. Collini , et al. 2004 Synovial sarcoma: a retrospective analysis of 271 patients of all ages treated at a single institution. Cancer 101:627–634. 15274077 10.1002/cncr.20386

[6] Lartigue, L. , A. Neuville , P. Lagarde , C. Brulard , P. Rutkowski , P. Dei Tos , et al. 2015 Genomic index predicts clinical outcome of intermediate‐risk gastrointestinal stromal tumours, providing a new inclusion criterion for imatinib adjuvant therapy. Eur. J. Cancer 51:75–83. 25466504 10.1016/j.ejca.2014.10.014

[7] Chibon, F. , P. Lagarde , S. Salas , G. Pérot , V. Brouste , F. Tirode , et al. 2010 Validated prediction of clinical outcome in sarcomas and multiple types of cancer on the basis of a gene expression signature related to genome complexity. Nat. Med. 16:781–787. 20581836 10.1038/nm.2174

[8] Nakagawa, Y. , K. Numoto , A. Yoshida , T. Kunisada , H. Ohata , K. Takeda , et al. 2006 Chromosomal and genetic imbalances in synovial sarcoma detected by conventional and microarray comparative genomic hybridization. J. Cancer Res. Clin. Oncol. 132:444–450. 16557383 10.1007/s00432-006-0089-5 PMC12161092

[9] Skytting, B. T. , J. Szymanska , Y. Aalto , T. Lushnikova , C. Blomqvist , I. Elomaa , et al. 1999 Clinical importance of genomic imbalances in synovial sarcoma evaluated by comparative genomic hybridization. Cancer Genet. Cytogenet. 115:39–46. 10565298 10.1016/s0165-4608(99)00095-3

[10] Lagarde, P. , J. Przybyl , C. Brulard , G. Pérot , G. Pierron , O. Delattre , et al. 2013 Chromosome instability accounts for reverse metastatic outcomes of pediatric and adult synovial sarcomas. J. Clin. Oncol. 31:608–615. 23319690 10.1200/JCO.2012.46.0147

[11] van der Graaf, W. T. , D. Orbach , I. R. Judson , and A. Ferrari . 2017 Soft tissue sarcomas in adolescents and young adults: a comparison with their paediatric and adult counterparts. Lancet Oncol. 18:e166–e175. 28271871 10.1016/S1470-2045(17)30099-2

[12] Chakiba, C. , P. Lagarde , D. Pissaloux , A. Neuville , C. Brulard , G. Pérot , et al. 2014 Response to chemotherapy is not related to chromosome instability in synovial sarcoma. Ann. Oncol. 25:2267–2271. 25070544 10.1093/annonc/mdu362 PMC4207728

[13] Ferrari, A. , Y. Y. Chi , G. L. De Salvo , D. Orbach , B. Brennan , R. L. Randall , et al. 2017 Surgery alone is sufficient therapy for children and adolescents with low‐risk synovial sarcoma: a joint analysis from the European paediatric soft tissue sarcoma Study Group and the Children's Oncology Group. Eur. J. Cancer 78:1–6. 28391003 10.1016/j.ejca.2017.03.003 PMC5567853

[14] Guillou, L. , J. M. Coindre , F. Bonichon , B. B. Nguyen , P. Terrier , F. Collin , et al. 1997 Comparative study of the National Cancer Institute and French Federation of Cancer Centers Sarcoma Group grading systems in a population of 410 adult patients with soft tissue sarcoma. J. Clin. Oncol. 15:350–362. 8996162 10.1200/JCO.1997.15.1.350

[15] Gronchi, A. , S. Ferrari , V. Quagliuolo , J. M. Broto , A. L. Pousa , G. Grignani , et al. 2017 Histotype‐tailored neoadjuvant chemotherapy versus standard chemotherapy in patients with high‐risk soft‐tissue sarcomas (ISG‐STS 1001): an international, open‐label, randomised, controlled, phase 3, multicentre trial. Lancet Oncol. 18:812–822. 28499583 10.1016/S1470-2045(17)30334-0

[16] Italiano, A. , N. Penel , Y. M. Robin , B. Bui , A. Le Cesne , S. Piperno‐Neumann , et al. 2009 Neo/adjuvant chemotherapy does not improve outcome in resected primary synovial sarcoma: a study of the French Sarcoma Group. Ann. Oncol. 20:425–430. 19088169 10.1093/annonc/mdn678

[17] Guillou, L. , J. Benhattar , F. Bonichon , G. Gallagher , P. Terrier , E. Stauffer , et al. 2004 Histologic grade, but not SYT‐SSX fusion type, is an important prognostic factor in patients with synovial sarcoma: a multicenter, retrospective analysis. J. Clin. Oncol. 22:4040–4050. 15364967 10.1200/JCO.2004.11.093

[18] de Necochea‐Campion, R. , L. M. Zuckerman , H. R. Mirshahidi , S. Khosrowpour , C. S. Chen , and S. Mirshahidi . 2017 Metastatic biomarkers in synovial sarcoma. Biomark Res. 5:4. 28191313 10.1186/s40364-017-0083-x PMC5297148

[19] Brennan, B. , M. Stevens , A. Kelsey , and C. A. Stiller . 2010 Synovial sarcoma in childhood and adolescence: a retrospective series of 77 patients registered by the Children's Cancer and Leukaemia Group between 1991 and 2006. Pediatr. Blood Cancer 55:85–90. 20213848 10.1002/pbc.22453

[20] Stanelle, E. J. , E. R. Christison‐Lagay , J. H. Healey , S. Singer , P. A. Meyers , and M. P. La Quaglia . 2013 Pediatric and adolescent synovial sarcoma: multivariate analysis of prognostic factors and survival outcomes. Ann. Surg. Oncol. 20:73–79. 22878620 10.1245/s10434-012-2587-9

[21] Mezzelani, A. , L. Mariani , E. Tamborini , V. Agus , C. Riva , S. Lo Vullo , et al. 2001 SYT‐SSX fusion genes and prognosis in synovial sarcoma. Br. J. Cancer 85:1535–1539. 11720441 10.1054/bjoc.2001.2088 PMC2363950

[22] Lesluyes, T. , G. Perot , M. R. Largeau , C. Brulard , P. Lagarde , V. Dapremont , et al. 2016 RNA sequencing validation of the Complexity INdex in SARComas prognostic signature. Eur. J. Cancer 57:104–111. 26916546 10.1016/j.ejca.2015.12.027

[23] Sun, Y. , B. Sun , J. Wang , W. Cai , X. Zhao , S. Zhang , et al. 2009 Prognostic implication of SYT‐SSX fusion type and clinicopathological parameters for tumor‐related death, recurrence, and metastasis in synovial sarcoma. Cancer Sci. 100:1018–1025. 19385976 10.1111/j.1349-7006.2009.01134.x PMC11159520

[24] Bertucci, F. , P. Finetti , J. Ostrowski , W. K. Kim , H. Kim , M. A. Pantaleo , et al. 2012 Genomic Grade Index predicts postoperative clinical outcome of GIST. Br. J. Cancer 107:1433–1441. 22929880 10.1038/bjc.2012.390 PMC3494448

[25] Salas, S. , C. Brulard , P. Terrier , D. Ranchere‐Vince , A. Neuville , L. Guillou , et al. 2015 Gene expression profiling of desmoid tumors by cDNA microarrays and correlation with progression‐free survival. Clin. Cancer Res. 21:4194–4200. 25878329 10.1158/1078-0432.CCR-14-2910

[26] Lesluyes, T. , L. Delespaul , J. M. Coindre , and F. Chibon . 2017 The CINSARC signature as a prognostic marker for clinical outcome in multiple neoplasms. Sci. Rep. 7:5480. 28710396 10.1038/s41598-017-05726-x PMC5511191

[27] Schleiermacher, G. , I. Janoueix‐Lerosey , and O. Delattre . 2014 Recent insights into the biology of neuroblastoma. Int. J. Cancer 135:2249–2261. 25124476 10.1002/ijc.29077

[28] Vlenterie, M. , V. K. Ho , S. E. Kaal , R. Vlenterie , R. Haas , and W. T. van der Graaf . 2015 Age as an independent prognostic factor for survival of localised synovial sarcoma patients. Br. J. Cancer 113:1602–1606. 26554650 10.1038/bjc.2015.375 PMC4705887

